# Comparative Genomic Insights into Chemoreceptor Diversity and Habitat Adaptation of Archaea

**DOI:** 10.1128/aem.01574-22

**Published:** 2022-10-31

**Authors:** Guihong Cha, Yugeng Liu, Qing Yang, Liping Bai, Lei Cheng, Wei Fan

**Affiliations:** a Shenzhen Branch, Guangdong Laboratory of Lingnan Modern Agriculture, Genome Analysis Laboratory of the Ministry of Agriculture, Agricultural Genomics Institute at Shenzhen, Chinese Academy of Agricultural Sciences, Shenzhen, China; b Key Laboratory of Development and Application of Rural Renewable Energy, Biogas Institute of Ministry of Agriculture and Rural Affairs, Chengdu, China; c Department of Chemical Engineering, Guangdong Technion-Israel Institute of Technology, Shantou, Guangdong, China; Royal Netherlands Institute for Sea Research

**Keywords:** archaea, habitat adaptation, chemoreceptors, ligand-binding domains

## Abstract

Diverse archaea, including many unknown species and phylogenetically deeply rooted taxa, survive in extreme environments. They play crucial roles in the global carbon cycle and element fluxes in many terrestrial, marine, saline, host-associated, hot-spring, and oilfield environments. There is little knowledge of the diversity of chemoreceptors that are presumably involved in their habitat adaptation. Thus, we have explored this diversity through phylogenetic and comparative genomic analyses of complete archaeal genomes. The results show that chemoreceptors are significantly richer in archaea of mild environments than in those of extreme environments, that specific ligand-binding domains of the chemoreceptors are strongly associated with specific habitats, and that the number of chemoreceptors correlates with genome size. The results indicate that the successful adaptation of archaea to specific habitats has been associated with the acquisition and maintenance of chemoreceptors, which may be crucial for their survival in these environments.

**IMPORTANCE** Archaea are capable of sensing and responding to environmental changes by several signal transduction systems with different mechanisms. Much attention is paid to model organisms with complex signaling networks to understand their composition and function, but general principles regarding how an archaeal species organizes its chemoreceptor diversity and habitat adaptation are poorly understood. Here, we have explored this diversity through phylogenetic and comparative genomic analyses of complete archaeal genomes. Signaling sensing and adaptation processes are tightly related to the ligand-binding domain, and it is clear that evolution and natural selection in specialized niches under constant conditions have selected for smaller genome sizes. Taken together, our results extend the understanding of archaeal adaptations to different environments and emphasize the importance of ecological constraints in shaping their evolution.

## INTRODUCTION

**FIG 1:**
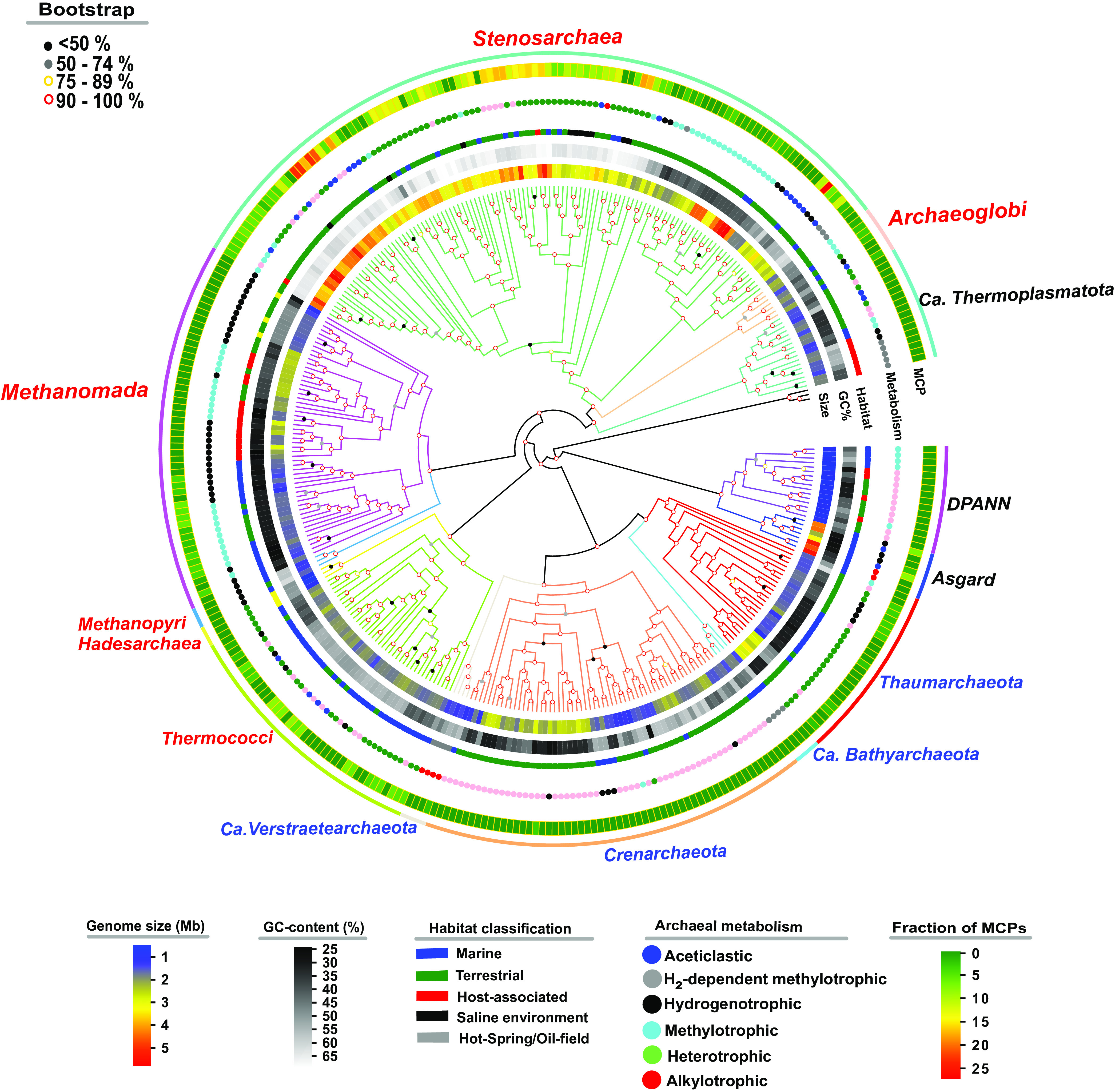
Phylogenetic tree of archaea. A maximum-likelihood phylogenetic tree was generated based on 122 archaeon-specific benchmarking universal single-copy orthologs (see Table S2) from 352 genomes (see Table S1). The group labels represent genome size, GC content, habitat classification, metabolism types, MCPs, and organism groups. Red and blue fonts indicate subgroups of the Euryarchaeota and TACK group, respectively, and the black font indicates the DPANN, Asgard, and *Candidatus* Thermoplasmatota groups. The bacterial kingdom formed the outgroup for the tree (black branch).

Archaea are considered one of the earliest life forms on earth (1). They occupy a key position in the tree of life and contribute a major fraction of microbial diversity (2). Currently, four major supergroups are recognized, designated Euryarchaeota, TACK (Thaumarchaeota, Aigarchaeota, Crenarchaeota, and Korarchaeota), Asgard, and DPANN (Diapherotrites, Parvarchaeota, Aenigmarchaeota, Nanohaloarchaeota, and Nanoarchaeota) (3). Here, the phylogeny tree distinguishes five archaeal superphyla: Euryarchaeota, Asgard, TACK, DPANN, and *Candidatus* Thermoplasmatota (Fig. 1; see also Fig. S1 in the supplemental material). Each of the supergroups includes several potentially phylum-rank clades (3–5), and its members are present in diverse environments, including oceanic, terrestrial, hot-spring, and oilfield habitats (6, 7).

Many archaeal and bacterial species have chemotactic abilities to sense signals regarding environmental gradients and use them to direct their movement toward more favorable locations (8–10). This process involves various signal transduction pathways, which are also called chemoreceptor-based signaling cascades or chemosensory systems (11–15). Archaeal chemotaxis has received less attention than the well-explored bacterial chemotaxis systems, but chemoreceptors called methyl-accepting chemotaxis proteins (MCPs) sense environmental signals and then drive chemotactic responses in conjunction with other components in both bacteria and archaea (16, 17). It is also known that despite variants of the chemotaxis signaling system among bacterial and archaeal species (18), there is substantial conservation of both the overall mechanism and proteins involved (10). Previous studies on the model organism Halobacterium salinarum have shown that MCPs might contribute to the adaptation of archaea to their habitats. This model halophilic organism displays tactic behavior and has chemoreceptors that sense a multitude of environmental stimuli, such as various chemicals (e.g., acetate and some amino acids), oxygen, osmolarity, and light (8, 18, 19). They regulate the autophosphorylation activity of the histidine kinase CheA, through the coupling protein CheW (20, 21). After autophosphorylation, a phosphoryl group is transferred from CheA to the response regulator CheY (17, 22). Phosphorylated CheY interacts with the archaea-specific chemotaxis protein CheF (23), and the CheF/CheY complex can then change the direction of rotation of the archaellum filament through interaction with the ArlCDE complex of the euryarchaeal archaellum motor (21, 24–31). Important structural components of the filament (both “major” and “minor”) include ArlA and ArlB archaellins (29, 32–36).

Chemoreceptors have two principal modules: input and output (37). The input module is usually composed of a single domain, although it has two or more domains in some chemoreceptors (38). The output module has a conserved structure, consisting of a long dimeric four-helix bundle composed of two symmetric antiparallel coils, which comprises the cytoplasmic signaling methyl-accepting (MA) chemotaxis-like domain (20, 39). In transmembrane chemoreceptors (the most common membrane topology class) input-output signaling is mediated by a HAMP linker (**H**istidine kinases, **A**denylate cyclases, **M**ethyl-accepting chemotaxis proteins, or **P**hosphatases) domain (20, 40). Chemoreceptors can recognize signals via their input module in several ways (8). A common mode of sensing is through the direct binding of effectors to the input ligand-binding domain (LBD) (20, 39, 41). Some input domains have cofactors, such as heme, which enables chemoreceptors to recognize oxygen (14). For example, Htr8 and Htr10 (HemAT) are required for attraction and phobic responses to oxygen of the archaeon *H. salinarum* (14). Although the structural composition of chemoreceptors is variable, the secondary structure of the MA domain, which interacts with the CheW adaptor protein and CheA histidine kinase, is highly conserved (41, 42), at least in taxa investigated to date. However, significant studies of the archaeal chemoreceptors remain confined to a few archaea: *H. salinarum* (19), Methanocaldococcus jannaschii (8), Methanosaeta harundinacea (13), Thermococcus kodakarensis (11), and recently Haloferax volcanii (33). Thus, further information about chemoreceptor diversity, evolution, and roles in habitat adaptation is required.

In efforts to obtain such information, we applied comparative genomic analysis using well-annotated archaeal phylogeny to explore chemoreceptor diversity in strains isolated from the six types of habitats in which archaea are most prevalent: terrestrial, marine, saline, host associated, hot spring, and oilfield (Fig. 1). We found that genomic size variations among archaea are associated with habitat adaptation. We observed a strong correlation between the GC contents of the genomes and their phylogenetic positions, with strains located within clusters having similar GC contents. In addition, archaea of similar habitats and metabolic characteristics are generally clustered in the tree and separated by small numbers of branches, but they are relatively scattered in terms of overall evolutionary relationships. We also noticed no indication of any pattern in the genome size in the tree or correlation with evolutionary relationships. Thus, many characteristics of archaea do not appear to be strongly linked to these evolutionary relationships, raising questions about their diversity, which we sought to elucidate by studying the diversity of their chemoreceptors and dynamic changes in them. We focused on MCP diversity in the analysis to identify the major classes of archaeal MCP domains and to discover correlations between them and adaptation to various environments. That the archaea MCP repertoire evolved following patterns similar to that observed in bacteria (e.g., *Campylobacterota*) indicates that the more heterogenous conditions are in niches in which archaea swim, the more diverse the MCP repertoire (43, 44). Our findings indicate that the tight coupling of signal identification by the LBDs and adaptative functions of the chemotaxis systems contributed to substantial diversity in chemotaxis mechanisms among archaeal groups.

## RESULTS AND DISCUSSION

### Diversity of chemoreceptors in archaea.

Archaea are widespread and occur in all environments on earth, where they comprise substantial portions of the microbial biomass, and they exhibit great diversity in metabolism, morphology, and physiology (3). MCPs are the first components of chemotaxis system to sense outside signals and initiate chemotactic responses in both bacteria and archaea (8). Therefore, we focused on chemoreceptors to elucidate their genomic evolution and the mechanisms of their adaptation to different environments.

**FIG 2:**
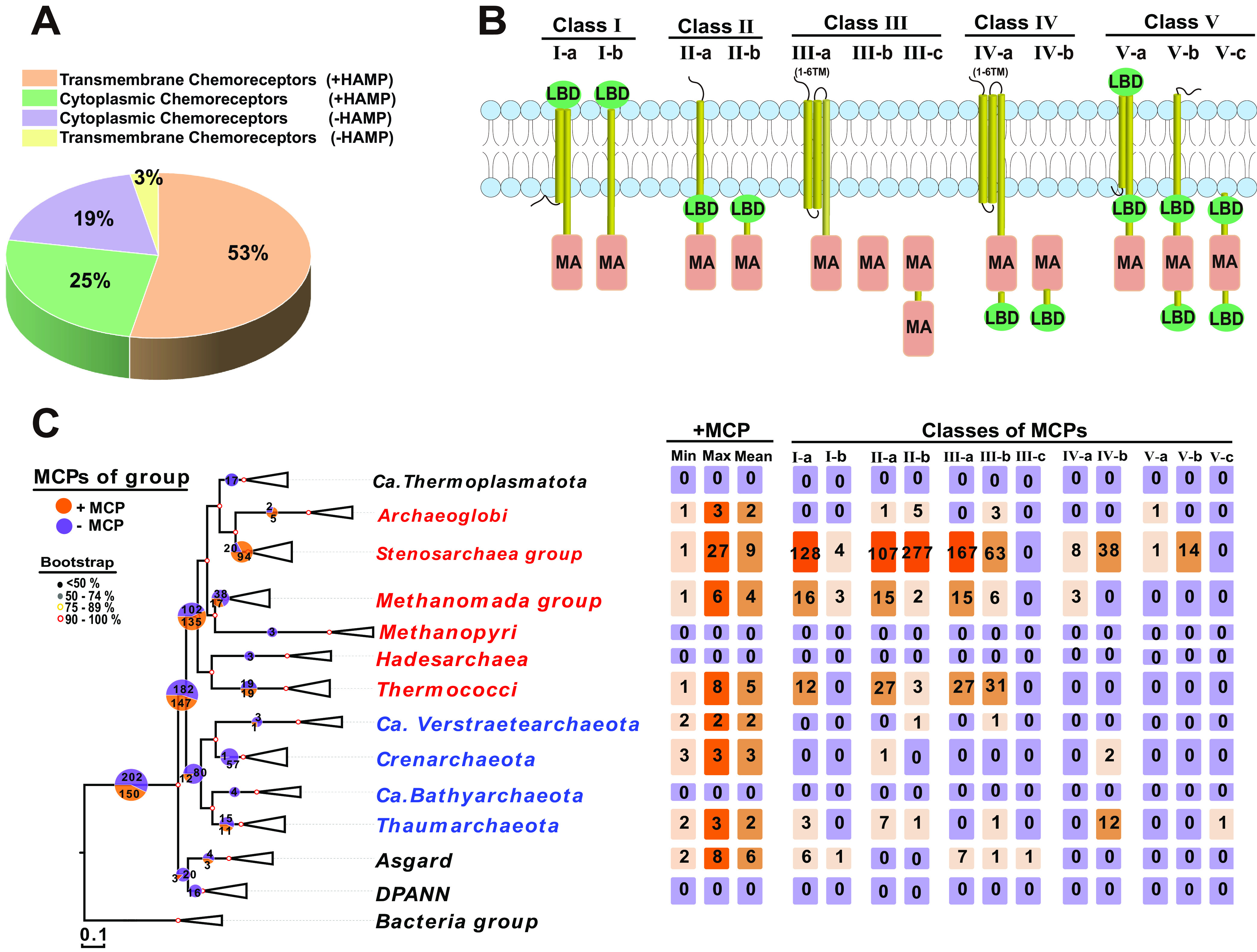
Chemoreceptor repertoires of archaea. (A) Abundances of four types of chemoreceptors in Archaea. “+HAMP” and “–HAMP” indicate MCPs that do and do not contain a HAMP domain, respectively. (B) MCP classification based on LBD location and membrane topology (see Table S3). LBDs are shown in green, and MA domains are shown in brown. The classification of MCP types depends on the location of LBDs, and there may be one or multiple LBDs at each location (see Fig. S2C). (C) Changes in numbers and types of MCPs during evolution. Red and blue fonts indicate subgroups of the Euryarchaeota and TACK groups, respectively, and the black font indicates the DPANN, Asgard, and *Candidatus* Thermoplasmatota groups. The bacterial kingdom formed the outgroup of the tree. The pie charts at nodes of the phylogenetic tree indicate the numbers of species in the branches. Light orange and light blue numbers indicate numbers of species with and without MCP genes, respectively; colored numbers in squares represent the quantities of MCPs and LBDs.

We searched all the chemoreceptors in the selected archaeal genomes and found that 150 of the 352 archaeal genomes have genes that encode MCPs and that 202 lack such genes (Fig. 1; see also Table S3 in the supplemental material). MCPs can be classified as either integral inner membrane proteins or soluble cytoplasmic proteins. Both cytoplasmic and membrane-bound MCPs reportedly have more heterogenous subcellular distributions than their bacterial counterparts, since archaeal arrays are frequently denser close to midcell positions (except by cell poles), whereas bacterial MCPs are more evenly distributed at the cell poles (11, 45). We found that 44% MCPs were cytoplasmic, and 56% were transmembrane (see Fig. S2A). We also observed that about 78% of chemoreceptors contain a membrane-proximal HAMP domain and that about 22% of the cytoplasmic chemoreceptors not contain HAMP in all their MCPs (Fig. 2A; see also Fig. S2B). HAMP domains are predicted to transmit signals detected by extracytosolic LBDs to the MA domains in cytoplasmic chemoreceptors, and we found that many of these receptors contain a HAMP domain in archaea (see Table S6). In previous studies, MCPs have been divided into four classes based on membrane topology and LBD location (20). Here, chemoreceptors were divided into five classes, each further divided into several subgroups based on LBD locations (Fig. 2B; see also Fig. S2C). Specifically, numerous cytoplasmic class V chemoreceptors contain a putative N-terminal LBD, and some also contain a C-terminal LBD (Fig. 2B; see also Table S5). The broad structural diversity of chemoreceptors that we detected, including in LBD locations and types, strongly suggests that these chemoreceptors could monitor wide arrays of signals from the cytoplasm and the environment.

Our phylogenetic tree also indicates that neither the most recently evolved group of archaea (“*Candidatus* Thermoplasmatota”) nor the ancient DPANN group has a chemotaxis system (Fig. 2C). Components of a chemotaxis system were mainly found in the Euryarchaeota, TACK, and Asgard superphyla (see Table S6). In addition, most of the archaeal phyla that have MCPs include some strains that lack MCPs. Moreover, the number of MCPs in members of the Stenosarchaea group varies from 1 to 27 (Fig. 2C; see also Table S3). The MCP repertoire is extremely limited in some species, but much broader in other species. In addition, the changes number or diverse of MCPs’ are not well conserved along branches of the evolutionary tree (Fig. 2C). We also show that all MCP-containing archaeal species have chemoreceptor type 44H as the dominant signal input (see Table S4 and Fig. S4). Unlike in *Bacteria* (11, 22, 43, 44), the chemotaxis system then evolved largely vertically in *Archaea* (11, 20, 46); these results also indicate that the chemotaxis system did not evolve solely in this manner in archaea and that low horizontal-gene-transfer (HGT) frequencies contributed (11).

### Enriched chemoreceptors in a specific habitat.

**FIG 3:**
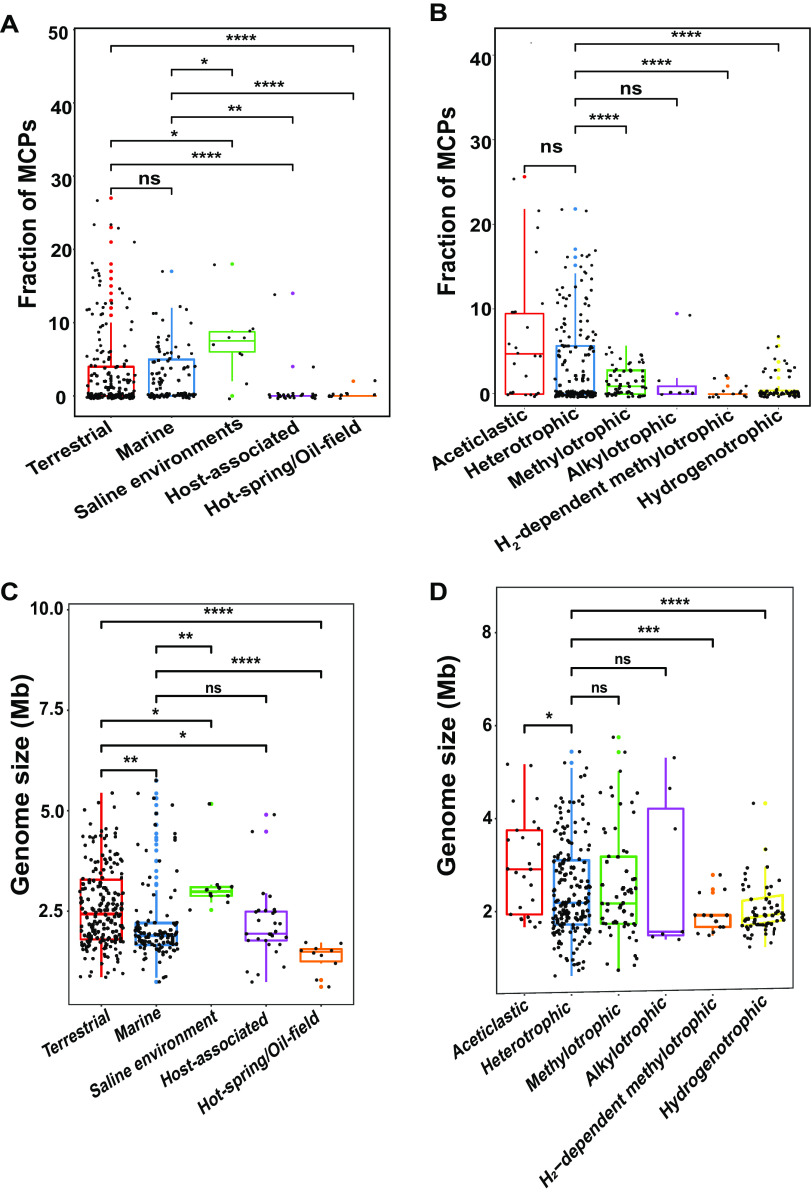
MCPs are involved in habitat adaptation. (A to D) Boxplots of archaeal MCP diversity in five biotopes (A), metabolic types in six biotopes (B), and genome size, as affected by niches (C and D). In each case, the median is shown in the box as a thick bar (unless it coincides with the borderline). The 25th and 75th percentiles are, respectively, in the lower and upper bounds of the box. Lines through the boxes indicate the minimum value and maximum values, while the whiskers correspond to the 1.5 interquartile range from the bounds. Outliers are marked by dots. Asterisks indicate significant differences between factors inferred from *t* tests and Student tests (*, *P* < 0.05; **, *P* < 0.01; ***, *P* < 0.001; ****, *P* < 0.0001; ns represent no significant difference).

To further study the relationship between the number of chemoreceptors and the ecological habitat of the archaea, we collected information on the niche of each strain and divided them into ecological groups. The results show that MCPs are present in most saline and terrestrial archaea, but fewer are present in host-associated, marine, thermal-spring, and oilfield archaea (Fig. 3A). These results suggest that the presence of MCPs in saline and terrestrial archaea enables them to move toward more optimal substrates and thereby cope more readily with changes in habitat conditions. In terms of metabolic types, we also observed that the numbers of MCPs were significantly richer in acetoclastic archaea than in hydrogenotrophic, methylotrophic, H_2_-dependent methylotrophic, heterotrophic, and alkylotrophic archaea (Fig. 3B). As in bacteria, the data for *Archaea* identified here are similar to what has been shown for *Bacteria*, namely, that genome size and niches with changing conditions and multiple gradients correlate with larger genomes, more diverse metabolisms, and more MCPs (43, 44). Collectively, the results mentioned above indicate that the number of MCPs encoded by archaeal genomes is closely related to their habitats.

### Variations in habitats account for the diversity of LBDs.

**FIG 4:**
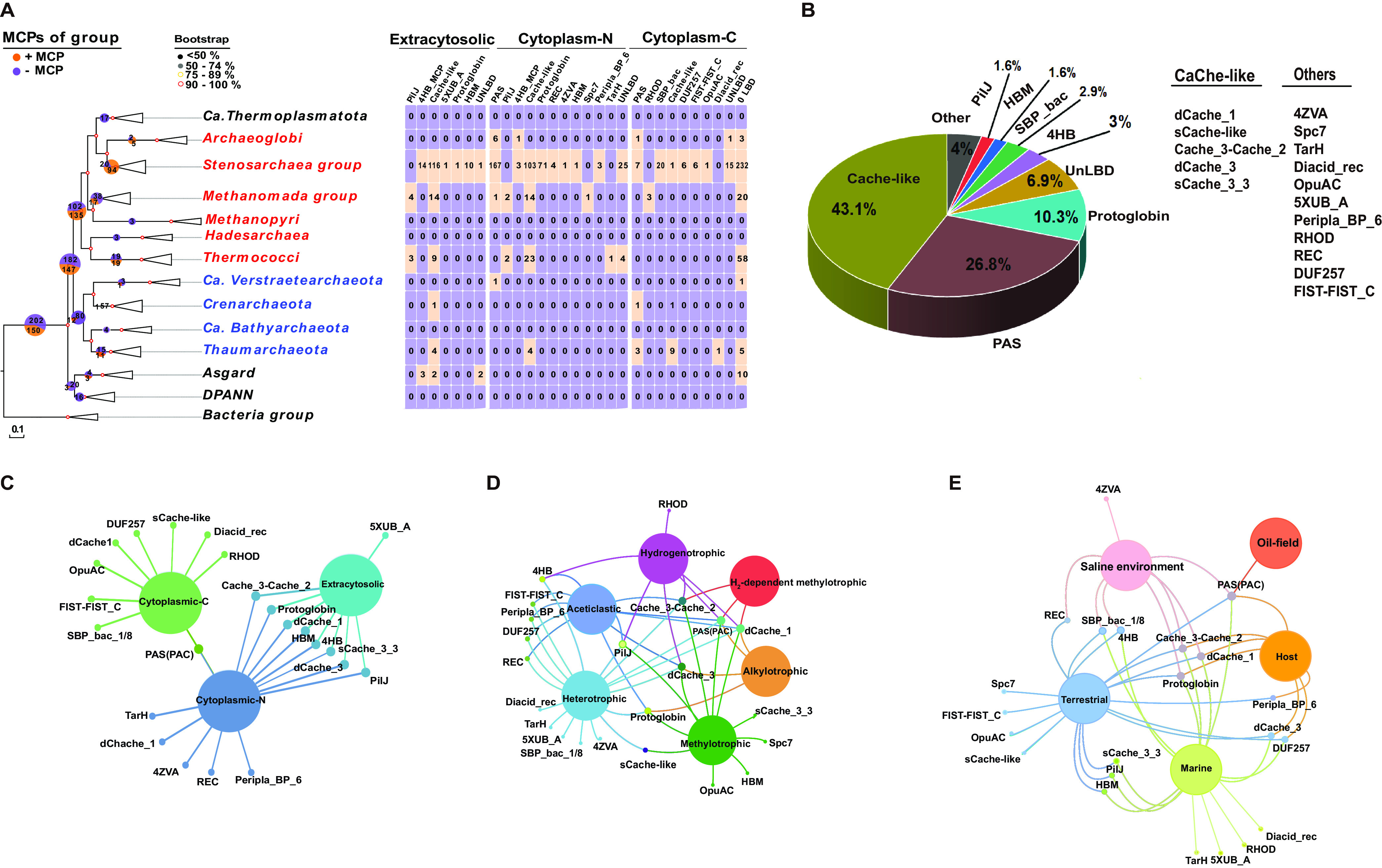
LBD diversity is shaped by ecological habitats. (A) LBD diversity and distribution. Colored numbers in squares represent the abundance of LBDs in archaeal genomes. (B) Relative abundances of the indicated LBD types in chemoreceptors. See Table S5 for details. (C) Venn network for three LBD locations. The green, blue, and light blue nodes represent sets of LBDs of cytoplasmic C, cytoplasmic N, and extracytosolic chemoreceptors. Nodes in the middle are shared by the muti-sets. Nodes connected with two edges are shared by the indicated pairs of sets. Outer nodes with only one connected edge are exclusive to the indicated sets. “N-terminal” and “C-terminal” indicate the percentages of chemoreceptors with the indicated domain on the amino-terminal and carboxy-terminal sides of the MA domain, respectively. (D) Venn network for six metabolism types. The dark green, mint green, red, purple, light blue, and orange circles represent sets of archaea with methylotrophic, acetoclastic, H_2_-dependent methylotrophic, hydrogenotrophic, heterotrophic, and alkylotrophic metabolism. Nodes in the middle are shared by the six sets. Nodes connected by two edges are shared by the indicated pairs of sets. Outer nodes with only one connected edge are exclusive to the indicated muti-sets. (E) Venn network for five habitat classifications. The light blue, yellowgreen, brown, pink, and orange circles represent, respectively, terrestrial, marine, host-associated, saline, and oilfield. Nodes in the middle are shared by the muti-sets. Nodes connected by two edges are shared by the indicated pairs of sets. Outer nodes with only one connected edge are exclusive to the indicated sets.

Chemoreceptors have multiple protein domains, most of which are structurally conservative, but not the LBDs. More than 80% of LBDs in chemoreceptors with known LBDs are members of the protoglobin, cache-like, and PAS/PAC families, while the PilJ, HBM, SBP_bac, and 4HB families account for about 9% (Fig. 4A and B; see also Table S5). Cache-like families, which include several types, are the most prevalent LBDs in chemoreceptors of the archaea-like stenosarchaea group (Fig. 4A and B; see also Table S5). PAS/PAC domains are the second most abundant family (Fig. 4B). These domains are found almost exclusively in cytoplasmic chemoreceptors (Fig. 4C), and many family members contain flavin adenine dinucleotide (FAD) or heme involved in oxygen and redox sensing processes (37, 47). Protoglobin domain is the third most abundant type of LBD (Fig. 4A and B). In the archaeon *H. salinarum* this domain contains heme and mediates oxygen sensing (14). In addition, some chemoreceptors contain an unknown N-terminal or C-terminal potential LBD that is least 80 amino acids in length (Fig. 4A and B).

We also observed that LBDs were significantly richer in heterotrophic strains than in other metabolic types (Fig. 4D) and were present in the archaea of most terrestrial and marine environments. Moreover, the LBD types of host-associated, saline, and oil-field archaea are also present in those of terrestrial and marine environments, but some LBD types occur only in MCPs associated with specific ecological habitats (Fig. 4E). These results indicate that their LBD diversity is closely related to variation in ecological niches. At the same time, these results also reveal that archaea inhabiting very specialized niches have less need for chemotaxis since there may not be much competition for nutrients, etc. Although the chemotaxis system evolved largely vertically in archaea, these findings suggest that gene duplication and mutation are not enough to create such diversity and that HGT may also be involved in the MCP enrichment and diversification of LBD types in specific habitats. Protein fusion between different classes of MCPs may also contribute to the evolution of new types during gene duplication, as well as HGT processes (46, 48). Overall, these results indicate that successful archaeal adaptation to specific habitats is associated with the acquisition and maintenance of MCPs, which might enhance fitness.

### Correlation between MCP number and genome size.

Chemoreceptors are crucial components of a chemotaxis system (8), but other proteins (such as CheA, CheW, and CheY) are also needed for signal transduction (24). Since CheY is a response regulator with a single REC domain, and proteins with a single REC domain are present in diverse signal transduction systems, study of the CheY protein of the archeon Halobacterium salinarum showed that, as in bacteria, CheY is phosphorylated and the protein and its phosphorylation state are essential for the chemotactic behavior of archaea (22). Thus, we examined CheW, CheY, and CheA proteins in further analyses of archaeal chemotaxis systems. We also examined the ArlA and ArlB components of their archaellin systems, which accept signals from the chemotaxis system and induce changes in organism behavior (23, 29).

**FIG 5:**
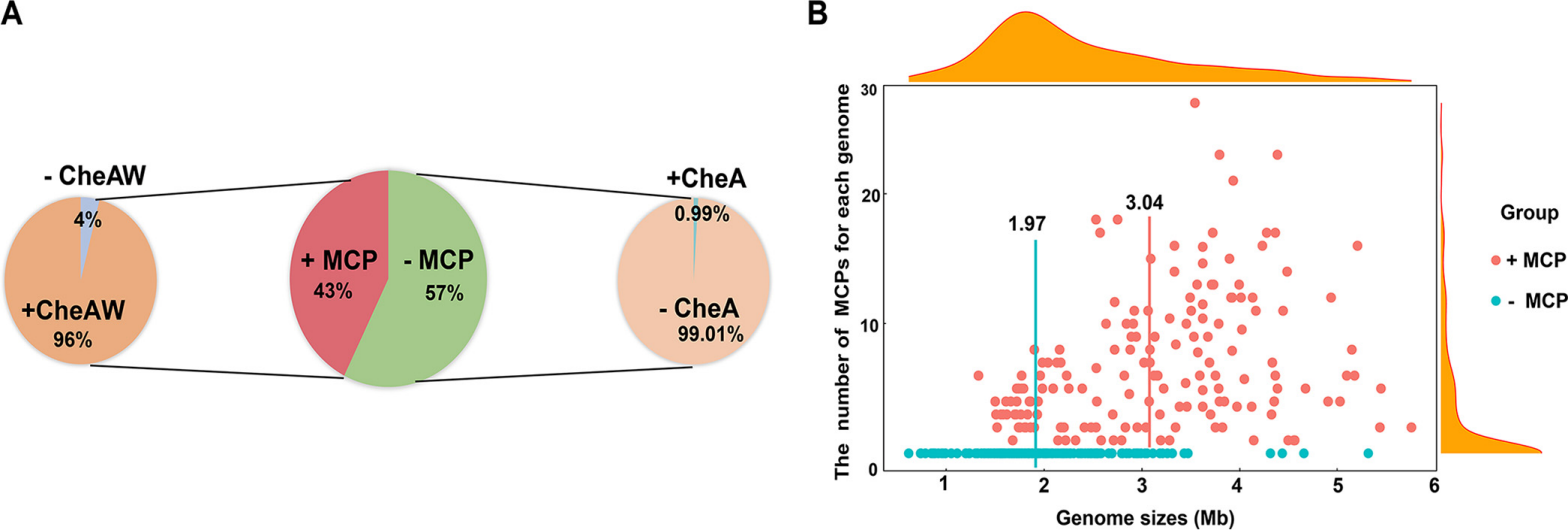
Correlation between MCP number and genome size. (A) Results of analysis of MCPs and important (CheA/CheW) component proteins. The middle, left, and right panels show, respectively, the abundance of MCPs in archaea, the abundance of CheA/CheW in strains with MCPs, and the abundance of CheA in strains with no MCPs. “+MCPs” and “–MCPs” indicate strains that do and do not contain MCP, respectively. (B) Relationship between the number of MCPs and the genome size, which varies from 0.49 to 5.75 Mb. The red dots represent strains with MCPs, and the red line represents their median number; the cyan dots represent strains that lack MCPs, and the cyan line represents their median number.

We found that nearly half (43%) of the analyzed strains contain MCPs, that most of these strains encode CheA, CheY, and CheW, and that only 4% of them do not encode CheA or CheW (Fig. 5A; see also Tables S6 and S7). In contrast, very few of the MCP-free strains encode CheA, and none encode CheW, which has no role when CheA is missing (Fig. 5A; see also Fig. S3). We also observed that few archaeal strains contain ArlA and ArlB (see Fig. S3). We postulated two hypotheses based on these results: (i) gene families of the chemotaxis and archaellin systems may have been lost or extended, and (ii) the chemotaxis system may play a key role in the regulation of not only motility patterns but also other systems, e.g., pili, in archaea.

Since the signal networks are complex and involve numerous signal transduction-related genes, and complex of signal networks maybe correlate with genome size. Thus, we also explored the potential relationship between the numbers of MCPs and genome size, which varies from 0.49 to 5.75 Mb (see Table S6). We found that MCP-free strains tend to have smaller genomes than do MCP-containing strains (Fig. 5B). MCP-containing strains have CheA, CheW, and other components of the chemotaxis system, as well as other systems, such as the archaellin system required for the archaellum filament. Next, we tested the possibility that the sizes of archaeal genomes have been affected by the extremity or heterogeneity of their habitats, which may clearly affect the diversity of many pathways involved in microbes’ metabolic capabilities, stresses, growth, and development. We found that strains growing in relatively extreme ecosystems had smaller genome sizes and fewer MCPs than in mild environments (Fig. 3C and D). For example, the number of archaeal MCPs appears to have been affected by habitat type and correlates with genome size (Fig. 1; see also Table S1). These results indicate that genome size correlates with the diversity and numbers of these systems and networks. In other words, the evolution of *Archaea* in specialized niches with more stable and constant conditions (e.g., host associated) has selected for *Archaea* with smaller genome sizes, streamlined and likely specialized metabolisms, and signal transduction systems.

### Conclusions.

We found differences in the numbers of MCPs encoded by the genomes of terrestrial, marine, saline, host-associated hot-spring, and oilfield archaea. More importantly, MCPs, such as the sensing substrate, were found to be involved in key processes, suggesting that they play major roles in the survival and adaptation of archaea in specific habitats. These findings show that signaling and adaptation processes are tightly coupled with the LBD and that show that evolution sensing and natural selection in specialized niches under constant conditions has selected for smaller genome sizes. Taken together, these results extend our understanding of archaeal adaptations to different environments and emphasize the importance of ecological constraints in shaping their evolution.

## MATERIALS AND METHODS

### Data sources.

Representative species of various archaea phyla with completely sequenced genomes were obtained from the NCBI RefSeq or GenBank database, and their taxonomic ranks were based on the NCBI taxonomy database (43). One strain was selected to represent each species with multiple completely sequenced strains. To cover some species that reportedly play an important role in extreme niches and to ensure genome quality, several incomplete but nearly complete genomes were included for analyses (see Table S1). Information on all the selected genomes (352 in total) was downloaded from the NCBI genome database (https://www.ncbi.nlm.nih.gov/genome/microbes/).

### Bioinformatics and data analysis software.

Phylogenetic trees were constructed by FastTree 2.0 (49). All homologous protein searches were conducted using BLAST 2.12.0 against local genomes (50), and MAFFT was used, with default parameters, for multiple sequence alignments (MSAs) (51). The topologies and domain architectures of all proteins were predicted by SMART, CDvist, and HHpred (52–54). Phylogenetic trees were modified and processed using EvolView (https://www.evolgenius.info/evolview/#login). Data were analyzed and figures were generated by using the Rstudio tool with the ggplot2, gigextra, tidyverse, hrbrthemes, and Viridis packages. The significance of differences among groups was assessed using a Student *t* test, implemented in Rstudio, and Venn networks were generated by software accessed through the Evenn website (http://www.ehbio.com/test/venn/#/).

### Phylogenetic analysis.

To construct a phylogenetic tree from the 352 archaeal genomes downloaded from the NCBI database (see Table S1), MSAs were created through concatenation of 122 phylogenetically informative protein- or protein domain-encoding sequences compiled in the Pfam v27 or TIGRFAMs v15.0 databases (55, 56), The 122 archaeal marker proteins (see Table S2) were selected using previously reported criteria (57, 58). A maximum-likelihood tree was constructed by FastTree v2.0 under the JC model, based on 16S rRNA gene sequences (~1,500 bp) obtained from annotated files of the 352 genomes in the NCBI Refseq Archaea genome database (https://ftp.ncbi.nlm.nih.gov/genomes/refseq/archaea/) or Silva database (https://www.arb-silva.de/). The 16S rRNA sequences were aligned by MAFFT 7.0 (59) and alignment positions with gap characters from >80% of the sequences were excluded by Gblocks 0.91b (60). A maximum-likelihood phylogenetic tree based on the alignments was constructed by FastTree v2.0 under the JC model, and bootstrap analysis was applied with 1,000 replications (49).

### Identification and analysis of protein components.

The genes encoding all chemoreceptors in the selected genomes were identified by BLASTP, and hits with an E value of <e^−3^ were assigned as candidates. The protein sequence of the MA domains of chemoreceptors from Methanomethylovorans hollandica DSM_15978 were selected as query sequences to perform the BLASTP analysis. All the candidates were reexamined for their domain organization by searching the SMART, HHpred, and CDvist databases manually (52, 54, 61). All chemoreceptor proteins are listed in Table S6. The genes encoding chemotaxis proteins (CheA, CheW, and CheY) and archaellin proteins (ArlA and ArlB) in the selected genomes were identified by BLASTP, and the hits with E value of <e^−3^ were assigned as candidates, but a threshold E value of 10 was applied for CheW and CheY. All of the candidates were reexamined for their domain organization by searching the SMART, HHpred, and CDvist databases manually (52, 54, 61). All identified proteins are listed in Table S6. For CheY, we chose sequences that were <200 amino acids in length and only had a receiver (REC) domain; all of the CheY candidate proteins are shown in Table S7.

### Classification of MCPs based on LBD.

The chemoreceptors were classified into different five classes based on the LBD locations, and each type was further divided into several subgroups based on their membrane topology. MCPs was divided into different H types (such as 44H, 40H, 28H, etc.) by Alexander and Zhulin (41) according to the sequence length and conservation of the MA domain in MCPs. All H types for MCPs in each genome were obtained from the MiST 3.0 database (61). The classifications of chemoreceptors are summarized in Table S4.

### Sample collection and analysis.

Archaea of diverse habitats and metabolic types were included in the analysis. Strain niches were characterized based on their isolation sites, which were determined manually by searching the NCBI Biosample (https://www.ncbi.nlm.nih.gov/biosample/?term=), DSMZ (https://iclac.org/databases/dsmz-str-profile-database/#), GDTB (https://gtdb.ecogenomic.org/), and ATCC (https://www.atcc.org/) databases (55). A Student *t* test was used to determine the significance of differences between groups of niches, and a Venn network was constructed by software accessed through Evenn (62; http://www.ehbio.com/test/venn/#/).
