# Supplementary material for: Comparative Genomic Insights into Chemoreceptor Diversity and Habitat Adaptation of Archaea
Source: Appl Environ Microbiol. 2022 Oct 31;88(22):e01574-22. doi: 10.1128/aem.01574-22 (PMC9680633; doi:10.1128/aem.01574-22)
Supplement: Supplemental file 1 — Fig. S1 to S4. Download aem.01574-22-s0001.pdf, PDF file, 0.7 MB [file aem.01574-22-s0001.pdf]

Supplementary Figure legends

FIG S1. **Results of phylogenomic analysis of archaea**. **(A)** Phylogenetic tree for 352 archaea species (**Table S1**) generated by FastTree based on concatenated sequences of 122 conserved proteins (**Table S2**). **(B)** Maximum-likelihood phylogenetic tree based on full-length 16S rRNA gene sequences of all type species of the archaea, using bacterial 16S rRNA sequences as an outgroup. Bootstrap values were calculated by non-parametric bootstrapping with 1000 replicates, and are represented by circles. Support values >90% are shown as red circles.

FIG S2. Results of analysis of MCPs' characteristics. **(A)** Abundances of cytoplasmic chemoreceptors and transmembrane chemoreceptors in Archaea. **(B)** Four types of transmembrane and cytoplasmic chemoreceptors are shown: the so-called class I, II, III, and IV chemoreceptor receptors, with two transmembrane regions (I and III) and two cytoplasmic regions (II and IV). The red asterisk indicates a domain (HAMP domain) that is not always present. Signal output coinciding with the MA domain in chemoreceptors is shown. Cytoplasmic chemoreceptors are shown close to the inner membrane, but in most cases do not interact with it directly. **(C)** MCP classification diagram. MCPs were divided into five classes based on their LBD location. Each position can exist in several LBD domains. This is a diagram of class II.

FIG S3. Results of analysis of the integrity of the chemotaxis system within an evolutionary framework. The group labels represent MCP, CheA, CheW, ArlA, and ArlB proteins, while dots and blanks indicate the presence and absence of genes encoding them, respectively. Colors of the branches indicate clusters of the archaea groups. Red and blue fonts indicate subgroups of the Euryarchaeota and TACK groups, respectively. Black font indicates DPANN, Asgard, and Candidatus Thermoplasmatota groups. The bacteria kingdom formed the outgroup (Black branch) of the tree.

Fig S4. **Changes in numbers and types of chemoreceptor types during evolution.** Red and blue fonts indicate subgroups of the Euryarchaeota and TACK groups, respectively. Black font indicates DPANN, Asgard, and *Candidatus* Thermoplasmatota groups. The bacteria kingdom formed the

outgroup (Black branch) of the tree. Light orange, light yellow and light blue numbers indicate numbers of species with and without chemoreceptor types (44H, 40H, and 28H), respectively Colored numbers in squares represent quantities of MCPs types.

Supplementary Tables

Table S1. Genomes used to construct the Archaea reference tree (**Fig. 1 and Fig. S1**) The first spreadsheet shows the number of genomes available and selected for the phylogeny, and the second spreadsheet shows NCBI accession numbers of the selected genomes.

Table S2. List of single-copy genes included in the multi-marker concatenation used to construct the archaea reference tree.

Table S3. MCP classification table.

Table S4. Summary of chemoreceptor types in archaea genomes.

Table S5. Domains identified in archaea chemoreceptors.

Table S6. Genomic size diversity and abundance of chemoreceptor in archaea.

TableS7. Summary of CheY homologs in archaea genomes.

Fig.S1

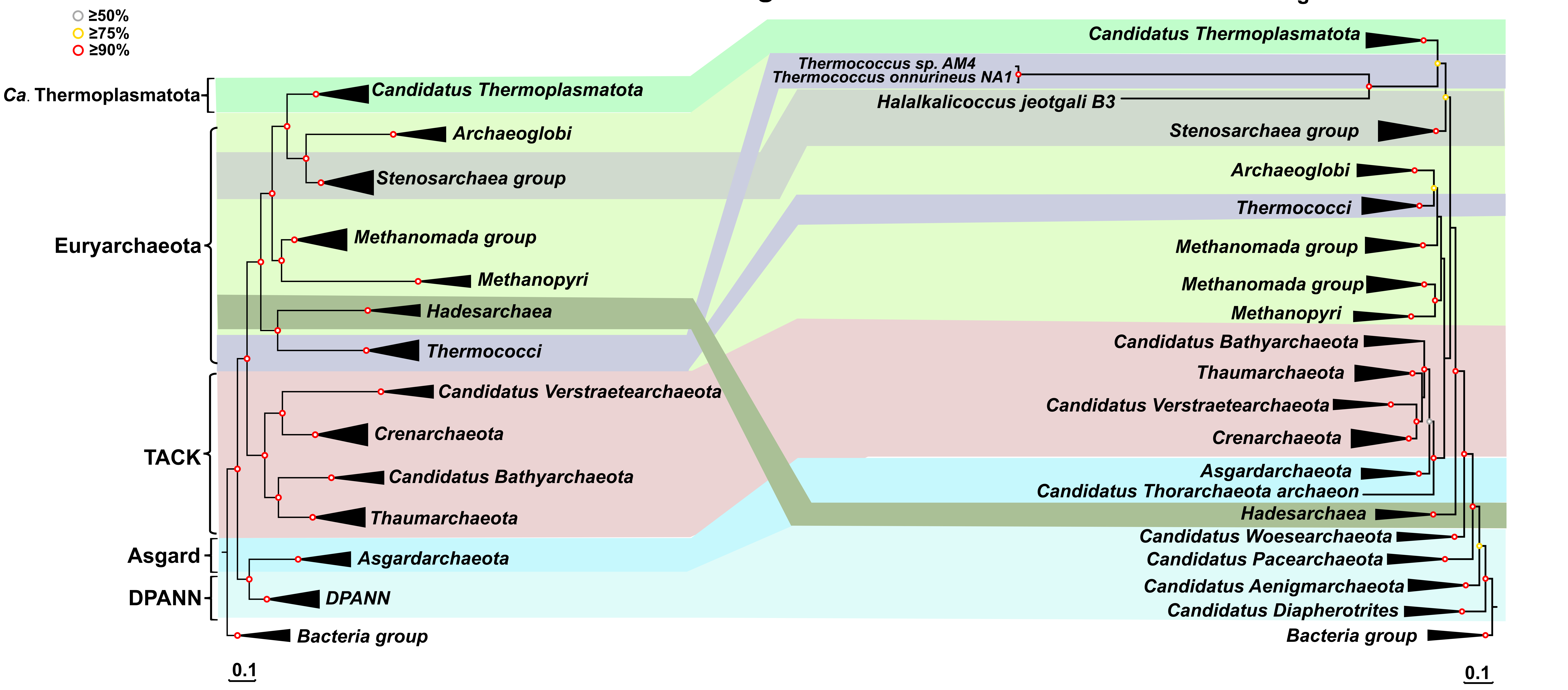

Genome classification
16S rRNA gene classification
≥50%
≥75%
≥90%
Ca. Thermoplasmatota
Euryarchaeota
TACK
Asgard
DPANN
Candidatus Thermoplasmatota
Archaeoglobi
Stenosarchaea group
Methanomada group
Methanopyri
Hadesarchaea
Thermococci
Candidatus Verstraetearchaeota
Crenarchaeota
Candidatus Bathyarchaeota
Thaumarchaeota
Asgardarchaeota
DPANN
Bacteria group
0.1
Candidatus Thermoplasmatota
Thermococcus sp. AM4
Thermococcus onnurineus NA1
Halalkalicoccus jeotgali B3
Stenosarchaea group
Archaeoglobi
Thermococci
Methanomada group
Methanomada group
Methanopyri
Candidatus Bathyarchaeota
Thaumarchaeota
Candidatus Verstraetearchaeota
Crenarchaeota
Asgardarchaeota
Candidatus Thorarchaeota archaeon
Hadesarchaea
Candidatus Woesearchaeota
Candidatus Pacearchaeota
Candidatus Aenigmarchaeota
Candidatus Diapherotrites
Bacteria group
0.1

Fig.S2

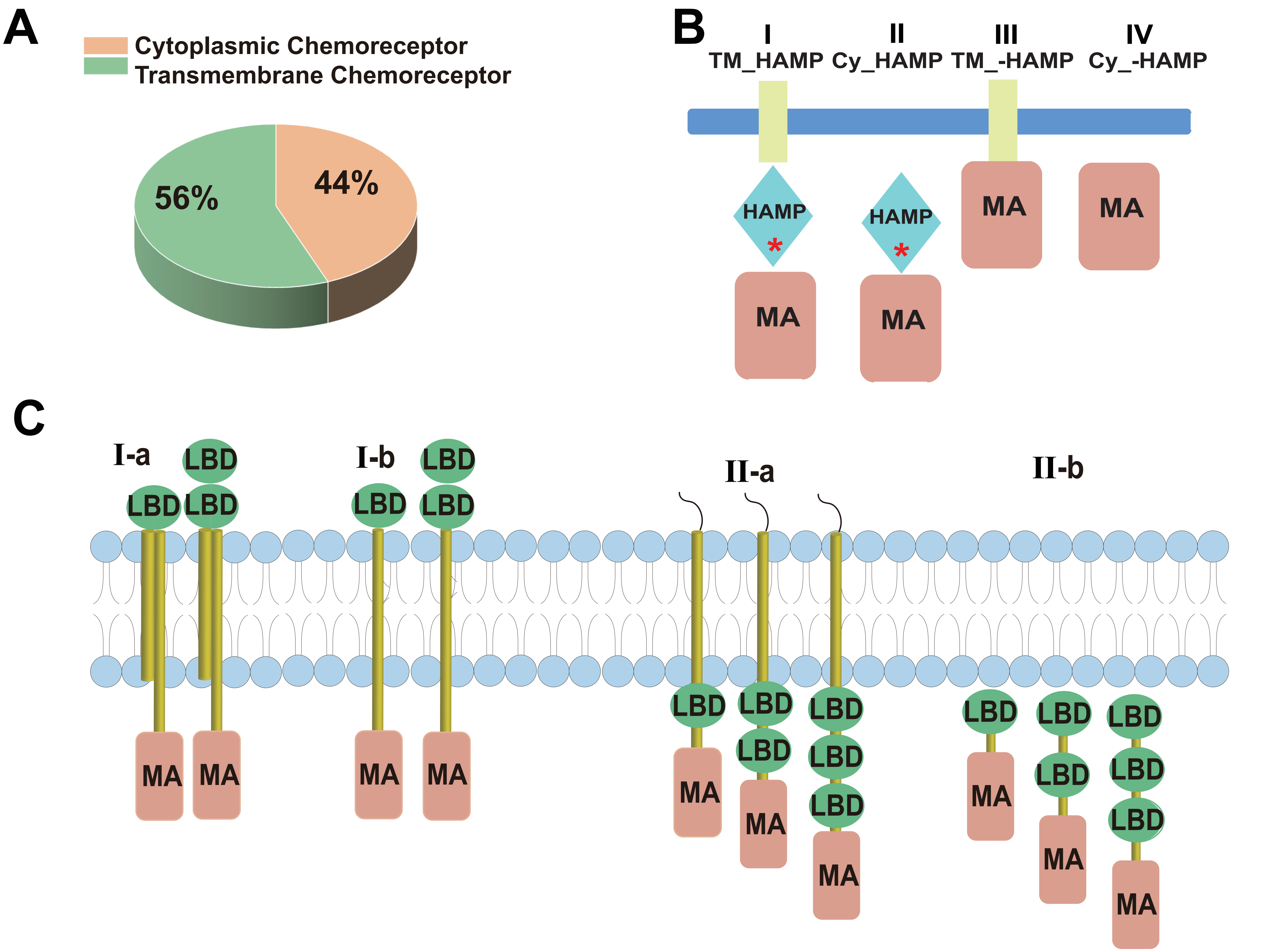
A
Cytoplasmic Chemoreceptor
Transmembrane Chemoreceptor
56%
44%
B
I
TM_HAMP
II
Cy_HAMP
III
TM_-HAMP
IV
Cy_-HAMP
HAMP
*
MA
C
I-a
I-b
II-a
II-b
LBD
MA

Fig.S3

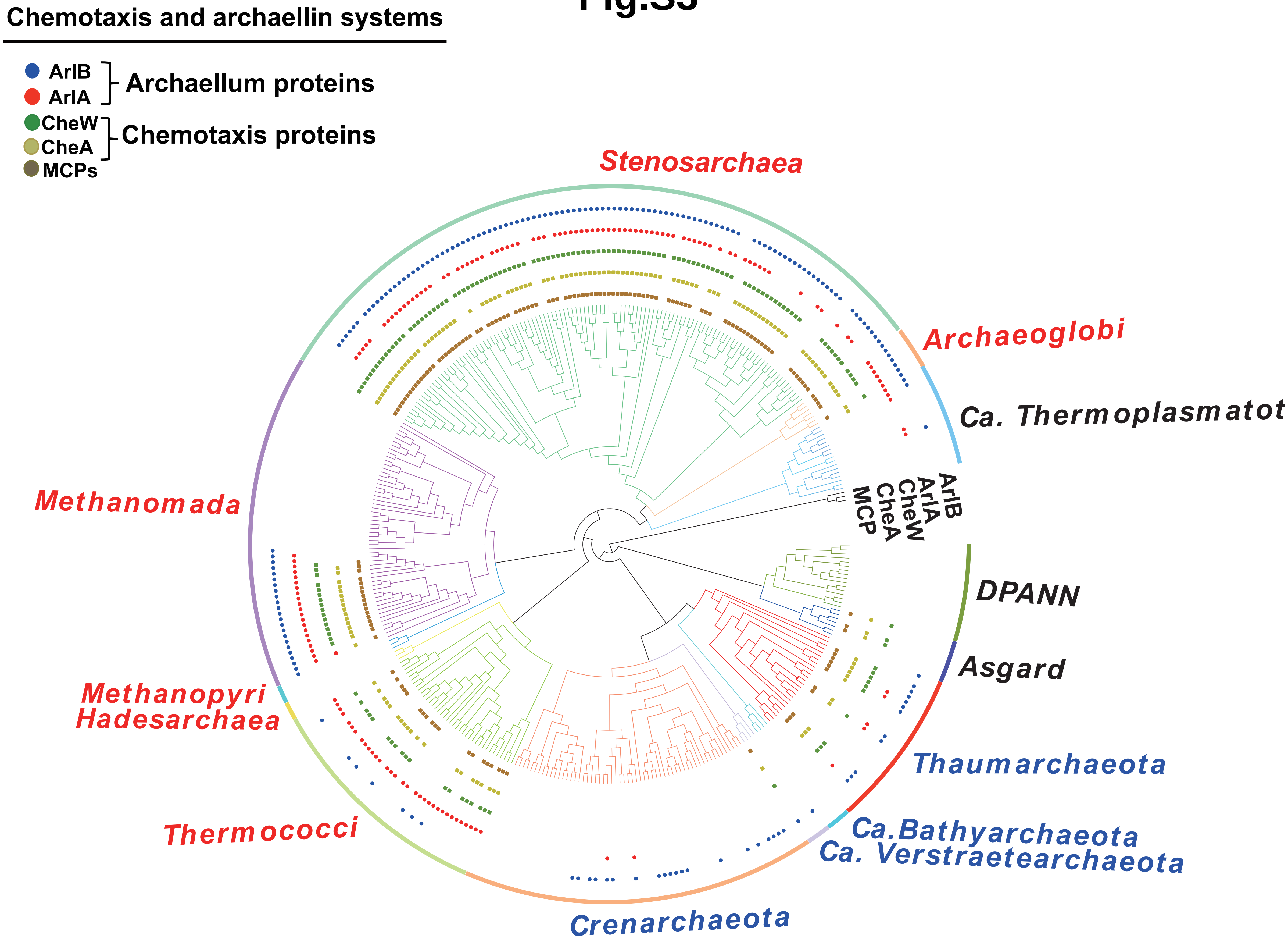

Note: Euryarchaeota (Red font); TACK group (Blue font); DPANN group, Asgard group and Candidatus Thermoplasmatota (Black font)

Fig.S4

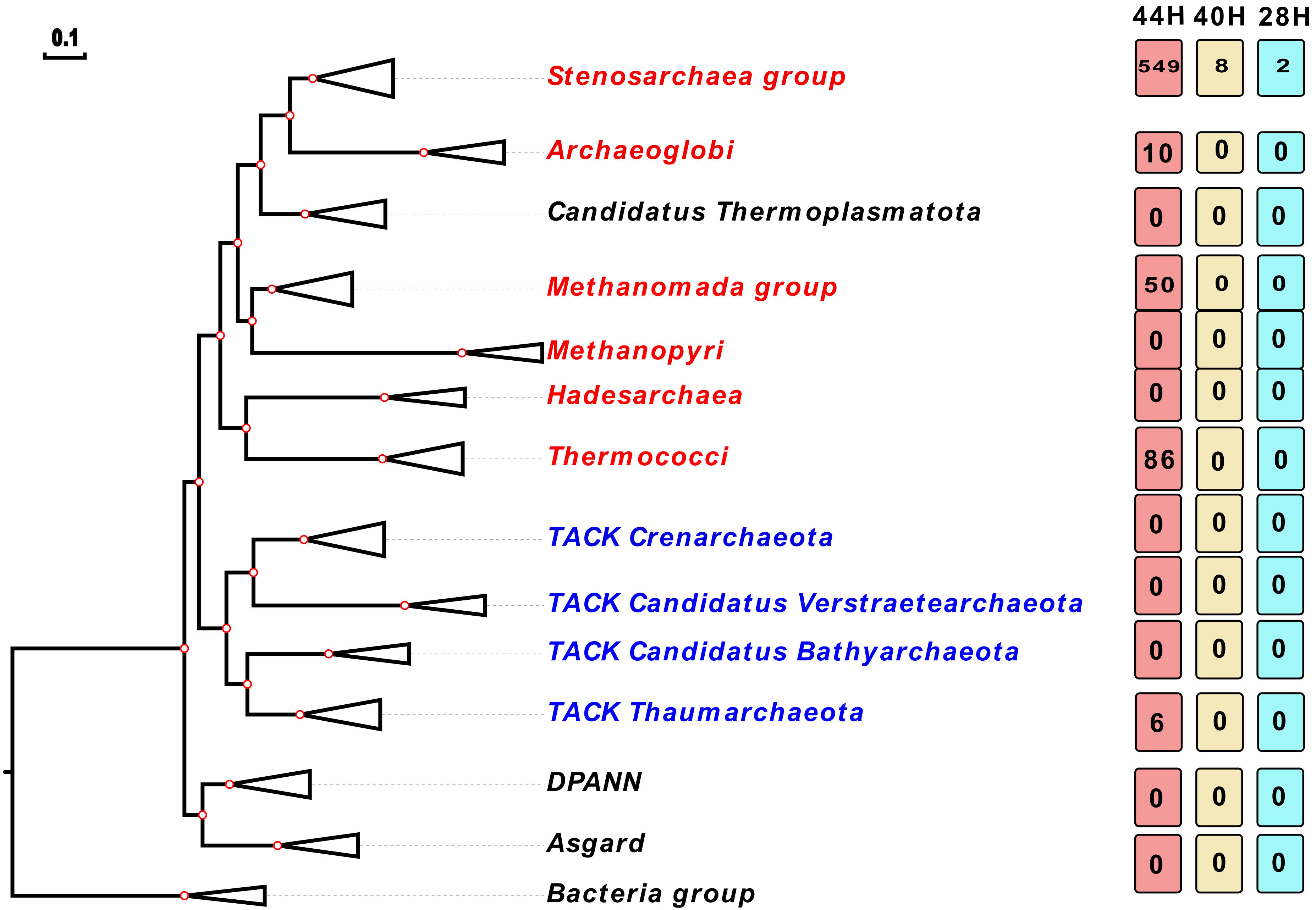
0.1
44H 40H 28H
Stenosarchaea group 549 8 2
Archaeoglobi 10 0 0
Candidatus Thermoplasmatota 0 0 0
Methanomada group 50 0 0
Methanopyri 0 0 0
Hadesarchaea 0 0 0
Thermococci 86 0 0
TACK Crenarchaeota 0 0 0
TACK Candidatus Verstraetearchaeota 0 0 0
TACK Candidatus Bathyarchaeota 0 0 0
TACK Thaumarchaeota 6 0 0
DPANN 0 0 0
Asgard 0 0 0
Bacteria group
